# Hierarchical hydrogen bonds enable stretchable, self-healing, and high-mobility polymer semiconductors

**DOI:** 10.1093/nsr/nwag277

**Published:** 2026-05-23

**Authors:** Antonio Facchetti

**Affiliations:** School of Materials Science and Engineering, Georgia Institute of Technology, USA

The field of organic electronics has evolved with the assumption that organic semiconductors are ductile; however, achieving flexible and particularly stretchable electronic materials and devices have been very challenging [1–4]. Polymer semiconductors that excel at charge transport are typically brittle, cracking easily under strain, and unable to recover from deformations. On the other hand, materials designed to be highly stretchable or self-healing often exhibit loosely π-stacked and dynamic morphologies that severely impede electronic carrier flow. Most existing solutions rely on adding elastomeric components, which considerably reduce the electronically active semiconductor phase [5], and by implementing single-stage hydrogen bonds [6,7]. Although these bonds help dissipate energy and repair damage, they rarely enable efficient interchain charge transport, nor do they offer the multilevel dynamic network needed to withstand large deformations. In a recent paper, Zhen's group and collaborators have introduced hierarchical hydrogen-bonded engineering that simultaneously achieves outstanding stretchability, notable self-healing ability, and high charge carrier mobility [8].

Zhen and coworkers incorporated a novel conjugation breaker, *N,-N'*-dicarbamoylpyridine-2,6-dicarboxamide (DCPDCA), into the backbone of diketopyrrolopyrrole-based semiconducting polymers (Fig. 1a). This unit creates two distinct hydrogen bond motifs with different strengths, affording a multilevel dynamic network: weaker bonds break first to dissipate strain energy, while stronger bonds maintain the overall connectivity and promote tight molecular stacking for efficient charge hopping. Single-crystal X-ray diffraction confirmed that DCPDCA molecules align nearly parallel to each other, forming two sets of intermolecular hydrogen bonds with remarkably high self-association constants (28.7 and 45.7), far exceeding those of conventional single hydrogen-bonded breakers. This parallel alignment and dual-bond system promote close π–π stacking that is essential for high mobility, while still allowing dynamic rearrangements under stress.

**Figure 1.**
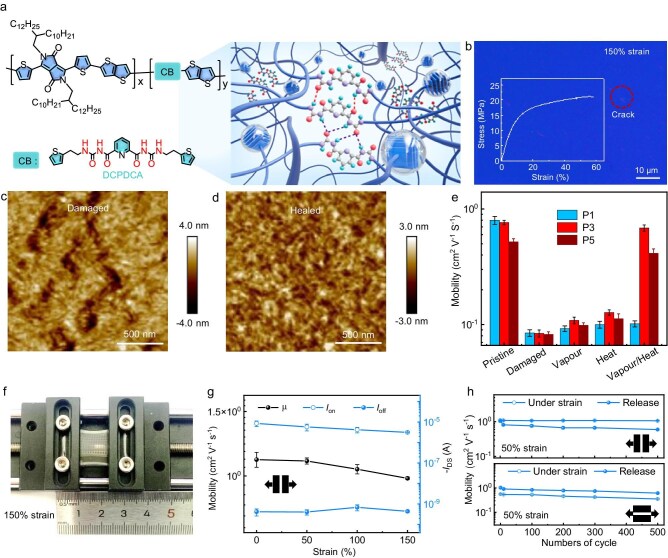
(a) Hierarchical hydrogen-bonded engineering for stretchable and healable polymers with a multilevel dynamic network in this work. (b) Optical microscopic image of P3 film transferred onto the polydimethylsiloxane substrate at 150% strain. The inset shows the stress–strain curve by employing the film-on-water technique. (c) Atomic force microscopy (AFM) image for the damaged film of P3. (d) AFM image for the healed film of P3. (e) Field-effect mobilities of damaged, postprocessed, healed transistor devices for P1, P3, and P5. (f) Images of the fully stretchable device under 150% strain. (g) Field-effect mobility, on-current and off-current as a function of various strains parallel to the charge transport direction. (h) Carrier mobility as a function of stretching–releasing cycles under 50% strain parallel and perpendicular to the charge transport direction.

The reference polymer with single-stage hydrogen bonds exhibited a crack onset strain of 100%, tensile modulus of 180.5 MPa and toughness of 0.15 MJ/m^3^ as revealed by optical microscopy and tensile testing. In stark contrast, the optimized terpolymer, P3 (containing 10 mol% DCPDCA), showed dramatically improved crack onset strain (150%), decreased elastic modulus (132.6 MPa), and 40-fold increased toughness (6.04 MJ/m^3^) as shown in Fig. 1b. After deliberately damaging the films to create nanocracks, the researchers exposed the film to a solvent-vapor and thermal treatments. Atomic force microscopy confirmed that cracks virtually disappeared in P3 films after treatment, leaving a smooth, continuous surface (Fig. 1c and d). P3 films recovered 90% of their original mobility, far better than the 78% recovery seen in the single hydrogen-bonded polymer P5 and the parent polymer P1 without hydrogen-bonded breakers (Fig. 1e). The key is the reversible nature of the two bond strengths: when the film is damaged, bonds break in a controlled way; during healing, both weak and strong bonds have a high probability of reforming, effectively ‘stitching’ the network back together.

Remarkably, the hierarchical hydrogen bonds did not sacrifice charge transport. Fully stretchable transistors made from P3 showed an initial hole mobility exceeding 1 cm^2^ V^−1^ s^−1^. Even under a strain of 150%, the mobility remained as high as 1.01 cm^2^ V^−1^ s^−1^ (Fig. 1f and g), which is an unprecedented value for intrinsically stretchable and healable semiconducting polymers. After 500 stretch–release cycles at a 50% strain, P3 transistors retained stable in both parallel and perpendicular directions (Fig. 1h). The devices also withstood poking, twisting, and biaxial stretching without significant performance degradation, proving their readiness for real-world mechanical challenges.

In summary, Zhen and coworkers provide a remarkable demonstration that hierarchical hydrogen bonds can be judiciously engineered into conjugated polymer backbones to address the long-standing trade-off between charge transport, stretchability, and self-healability. This approach could be extended to other conjugated polymer systems, paving the way for truly robust, skin-like electronics that repair themselves after damage while maintaining high performance under extreme mechanical deformations.

## References

[1] Chen J, Huang W, Zheng D et al. Nat Mater 2022; 21: 564–71. 10.1038/s41563-022-01239-9 35501364

[2] Wang Z, Liu Y, Zhao Y. Adv Mater 2025; doi: 10.1002/adma.202507519. 10.1002/adma.202507519

[3] Kang J, Tok JBH, Bao Z. Nat Electron 2019; 2: 144–50. 10.1038/s41928-019-0235-0

[4] Jang J, Choo H, Lee S et al. Nat Electron 2025; 8: 474–84. 10.1038/s41928-025-01389-z

[5] Xu J, Wang S, Wang G-JN et al. Science 2017; 355: 59–64. 10.1126/science.aah4496 28059762

[6] Oh JY, Rondeau-Gagné S, Chiu Y-C et al. Nature 2016; 539: 411–5. 10.1038/nature20102 27853213

[7] Yue H, Wang Y, Luo S et al. Sci Adv 2024; 10: eadq0171. 10.1126/sciadv.adq0171 39356754 PMC11446264

[8] Yue H, Wang Y, Meng Z et al. Natl Sci Rev 2026; 13: nwag162. 10.1093/nsr/nwag162 42238991 PMC13228146

